# Camera-based visual feedback learning aid for recovering sense of smell and taste in COVID-19 survivors: a proof-of-concept study

**DOI:** 10.3389/fpsyg.2023.1213254

**Published:** 2023-07-12

**Authors:** Veena Kumari, Satyam Chauhan, Krupa Vakani, Elena Antonova, Jacky Bryant

**Affiliations:** ^1^Division of Psychology, Department of Life Sciences, College of Health, Medicine and Life Sciences, Brunel University London, London, United Kingdom; ^2^Centre for Cognitive and Clinical Neuroscience, College of Health, Medicine and Life Sciences, Brunel University London, London, United Kingdom; ^3^Learning JBE Ltd, London, United Kingdom

**Keywords:** visual feedback, learning, smell, taste, COVID-19, impairment, intervention

## Abstract

**Introduction:**

A significant proportion of people report persistent COVID-19-related anosmia, hyposmia or parosmia, often accompanied with ageusia, hypogeusia or dysgeusia. Here, we present a proof-of-concept study that assessed the feasibility and acceptability of a new Camera-Based Visual Feedback Learning Aid (CVFLA) and explored its potential to restore or improve persistent COVID-19-related smell and/or taste impairment.

**Methods:**

Fifteen adult participants with persistent smell and/or taste impairment were randomly allocated to 7-, 14-, or 21-days baseline of symptom monitoring before receiving the intervention in up to 10 sessions (length and frequency determined by participant’s preference and progress) using a specialised CVFLA apparatus (patent no. 10186160). Smell and taste were assessed pre- and post-intervention subjectively, and also objectively using the ODOFIN Taste Strips and Sniffin Sticks. Participant feedback about their experience of receiving CVFLA was obtained via a semi-structured interview conducted by someone not involved in delivering the intervention.

**Results:**

The intervention was extremely well received, with no dropouts related to the intervention. There was also a significant improvement in smell and taste from pre- to post-CVFLA intervention (mean number of sessions = 7.46, *SD* = 2.55; total duration = 389.96 min, *SD* = 150.93) both in subjective and objective measures. All participants, except one, reported experiencing some improvement from the 2nd or 3rd session.

**Discussion:**

This new CVFLA intervention shows promise in improving COVID-19 related impairment in smell and taste with a very high level of acceptability. Further studies with larger samples are required to confirm its potential in restoring, improving or correcting smell and/or taste impairment in relevant clinical and non-clinical groups.

## 1. Introduction

A new onset of smell or taste loss has been considered a clinical indicator of SARS-CoV-2 infection since the start of the pandemic (e.g., Borsetto et al., 2020; Costa and Carnauba, 2020; Giacomelli et al., 2020; Lechien et al., 2020; Spinato et al., 2020). About 1 in 5 people with COVID-19 report persistent (i.e., lasting more than 10 days) COVID-19-related anosmia (loss of the sense of smell), hyposmia (reduced sense of smell) or parosmia (distorted sense of smell; e.g., Chary et al., 2020; Chiesa-Estomba et al., 2020; Antolín-Amérigo et al., 2021; Printza et al., 2021). A similar proportion of people with COVID-19 report ageusia (loss of the sense of taste), hypogeusia (reduced sense of taste) or dysgeusia (altered perception of taste), with many people reporting both smell and taste impairment (Wang et al., 2023). Even though the prevalence of COVID-19-associated smell and taste impairment decreased with later variants of the virus (Boscolo-Rizzo et al., 2022), it still appears significant for the Omicron variant at around 12% in people with European ancestry (von Bartheld and Wang, 2023). Furthermore, persistent qualitative disturbances of smell and/or taste have been reported in around one-third of patients who recover from COVID-19 (Ercoli et al., 2021).

An early longitudinal study (Boscolo-Rizzo et al., 2022) that followed up people with COVID-19 for eight weeks found that one in three people had smell or taste impairment at four weeks, and 1 in 5 still had smell and/or taste impairment when assessed at eight weeks, with that the loss of smell and taste being the most prevalent long-lasting symptom, followed by fatigue and breathing problems. Later studies (e.g., Jensen et al., 2022) also show impaired smell in about 20% of people at six-months post-COVID 19. Full recovery of smell and/or taste may occur by one year in about half of such cases (Nguyen et al., 2021; review, Peterson et al., 2021; Boscolo-Rizzo et al., 2023) but may still persist in a significant proportion even two years after the infection (Boscolo-Rizzo et al., 2023).

Impaired sense of smell and taste have implications for mood and daily activities of the affected individuals. Empirical evidence shows that pleasant and unpleasant smells are powerful manipulators of mood and emotions (e.g., Kaviani et al., 1998) and, perhaps not surprisingly, smell and/or taste impairment in the context of COVID-19 has been associated with low mood and anxiety (Dudine et al., 2021), unhealthy eating patterns (Javed et al., 2022), reduced quality of life and safety related issues (Coelho et al., 2021) as well as with brain fog (Garcia-Melendez et al., 2023). Even in non-COVID populations, impaired sense of smell is reported to occur in people with depression (Pause et al., 2001, 2005; Pollatos et al., 2007; Yuan and Slotnick, 2014) and has been linked with cognitive impairment and depression in the elderly, in certain types of dementias (Suzuki et al., 2004; Seo et al., 2009) and known to influence appetite and immunity (Schiffman and Graham, 2000). Thus, there is a need to find acceptable and scalable interventions that can aid recovering of smell and taste in the context of COVID-19 as well as in other disabling conditions that commonly present with impaired sense of smell and/or taste.

The present study was designed to assess the acceptability, feasibility and potential benefits of a specialized Camera-Based Visual Feedback Learning Aid (CVFLA) in restoring, improving and/or correcting the sense of smell and taste, along with possible changes in mental health and well-being, in people with persistent COVID-19-related smell and/or taste impairment. This CVFLA involves the use of a camera-based technology and a specialized collar technique for smell and taste training whereby real time video feedback about the individual is observed, while the direct view of the self is obscured (patent no. 10186160). During a session, the individual “learns” by observing their self through real time video feedback. In an early study by Ramachandran and Rogers-Ramachandran (1996), a series of patients were reported to recover phantom limb sensation using a technique involving a virtual reality mirror box (a mirror placed vertically on the table and reflected the patients’ intact hand superimposed on the experienced position of the phantom limb). There is recent evidence that visual feedback training can help to restore accurate sensation of the self, change sensations within the self (from discomfort to comfort and *vice-versa* as required), improve mobility, balance and movement, reduce pain, retrain stress responses, improve breathing, and many other sensations and pertaining to the individual (e.g., Deconinck et al., 2015; Kim and Lee, 2020; Pak and Lee, 2020). The specialized CVFLA we report had shown promise in unpublished case studies. The present proof-of-concept study aimed to examine the feasibility of delivering this intervention, its acceptability and potential to facilitate recovery of smell and taste that was lost or distorted due to COVID-19.

## 2. Methods

### 2.1. Participants

The study initially involved 16 adults residing in different parts of the UK who self-reported experiencing persistent COVID-19-related loss of smell and/or taste. Of these, 15 participants (5 males, 10 females; age range: 20–62 years) completed the study (one person could not continue for personal reasons). The participants were recruited through social media and contacts with relevant charities as well as from our ongoing COVID-19-related projects (Vakani et al., 2023). The study inclusion criteria required all participants to be (i) aged ≥18 years, (ii) experiencing persistent (lasting >10 days) smell and taste impairment following COVID-19 infection, and (iii) able to provide written informed consent.

The study was approved by the University Research Ethics Committee (ref no. 18771-LR-Oct/2019–20,701-1). All participants provided written informed consent and were compensated for their time and travel expenses. All study procedures followed ethical standards set by the Helsinki declaration (1964).

### 2.2. Design and procedure

The study utilized a non-concurrent multiple baseline across participants design (Watson and Workman, 1981). This is a type of single-case design where each participant acts as their own control, and can be used to study the effect of an intervention across several participants. When using this design, the intervention for any given problem or behavior begins at different times for the different participants; and effects of the intervention are shown when changes in the target problem/behavior are observed that coincide with the intervention and do not systematically covary with the duration of the baseline. For this study, we opted for a non-concurrent type to allow more flexibility in recruiting participants, especially when the pandemic-related restrictions in the context of laboratory-based research studies at the university were continuously changing. The study involved three different pre-selected baselines (7 days, 14 days, and 21 days), with an equal number of participants in a pre-determined sequence allocated to each of the three baselines to avoid experimenter bias (Christ, 2007).

Of 15 participants in the study, five participants had been allocated to receive the CVFLA intervention after 7 days, five participants after 14 days, and five participants after 21 days of baseline periods of monitoring for changes in the sense of smell and/or taste (see Table 1). However, one of the participants who had been allocated to start receiving the intervention after a 14-day baseline, started receiving the intervention a week later than planned due to personal reasons, and hence a 14-day baseline became a 21-day baseline in this case. Therefore, there were only four participants with a 14-day baseline monitoring and six participants with a 21-day baseline period in the final sample; this, if anything, contributes to the robustness of the results as a 21-day baseline was sufficiently long for smell and taste to return (but it did not happen) spontaneously without the CVFLA intervention.

**Table 1:** Study design and phases.

Screening	Baseline	Pre-intervention Assessments	CVFLA Intervention	Post-intervention Assessments
Demographics COVID history Smell and Taste impairment *If found to meet inclusion criteria – allocated to* 7, 14, or 21 days of smell and taste monitoring	7, 14, or 21 days of symptom (smell and taste) monitoring *If smell and taste impairment still present (self-reported) at the end of the allocated baseline period – invited for Pre-intervention Assessment*	Smell and Taste Mental health and well-being Interoceptive awareness	Up to 10 sessions over 5–10 weeks	Smell and Taste Mental health and well-being Interoceptive awareness Semi-structured interview to obtain participant feedback about their experience of the CVFLA

Prior to being allocated to 7/14/21 days of baseline (smell and taste monitoring), all participants were carefully screened to ensure they met our study inclusion criteria (see Table 1). In addition, information was obtained for any known allergies and medical history. The selected participants were asked to subjectively rate their sense of smell and taste during their allocated baseline period, and invited for the pre-intervention assessments if their smell and taste impairment persisted at the end of the allocated baseline period (found to persist in all cases) (Table 1).

For pre-intervention assessments, a trained researcher (SC or KV) administered a range of self-report measures to obtain information on participants’ COVID history, mental health and well-being, interoceptive awareness, smell and taste impairment, and objectively assessed their smell and taste impairment using an ODOFIN taste strip and Sniffin stick test kit (Rumeau et al., 2016). They then received the intervention (see “CVFLA Intervention” CVFLA intervention) and were re-assessed one week after the last intervention session on the same measures as used for pre-intervention assessments. All 15 participants provided subjective ratings of smell and taste impairment after the last intervention session (audio-video recordings obtained and the videos subsequently rated by someone who was not involved in delivering the intervention for scoring purposes), but four (three with significant travel commitments, and one re-infected with COVID-19) of the 15 participants did not complete the remaining post-intervention assessments.

### 2.3. Pre- and post-intervention assessments

Smell and taste, mental health and well-being, and interoceptive awareness were assessed before and after the intervention. In addition, a semi-structured interview was conducted at the very end of study participation (post-intervention) by a researcher who was not involved in delivering the intervention (KV) to gather participant feedback about the acceptability of the current version of the CVFLA and possible future improvements.

#### 2.3.1. Smell and taste

Smell and taste impairments were first assessed subjectively by asking the participants to rate their ability to smell (loss of smell and distorted sense of smell) and taste (loss of taste and distorted sense of taste) on a seven-point scale [“not at all” (0) to “very severe” (6)]. The ODOFIN *Taste and Sniffin Sticks* (Rumeau et al., 2016) were then used to measure smell and taste impairment objectively. The ODOFIN smell and taste identification test has 12 Sniffin sticks of different odors (orange oil, leather, cinnamaldehyde, peppermint oil, banana, lemon oil, anethole, coffee, clove oil, pineapple, rose, and fish) and four paper strips impregnated with salt, sugar, sour, and bitter taste. Each stick was presented with a gap of 5 s under three conditions (smelling with left, right, and both nostrils respectively). Each time, a cue card was presented with four options to sniff the stick and choose the option that matched their olfactory perception. They were asked to guess the smell if they could not smell anything. A total score was achieved for each condition by adding the individual response, with 0 indicating “no smell” and 12 indicating “maximum ability to smell”. Four taste strips were given with a gap of 30 s, and a cue card was presented with four different options. They were asked to choose the option that matched their taste perception. A score of 0 was given if the response was wrong, and a score of 1 was given if it was correct. All information, including prompted/unprompted answers, whether guessed, known, or remembered from the previous trial, distorted or no smell/taste, were recorded on a separate scoring sheet.

#### 2.3.2. Mental health and well-being

The levels of depression, anxiety and stress were assessed using the Depression Anxiety and Stress Scale (DASS-21; Lovibond and Lovibond, 1995). This 21-item self-report scale has three subscales (each with seven items): depression, anxiety and stress. Each item is rated on a four-point scale (0 to 3) based on how often in the past week it applied to them. Higher scores indicate higher levels (severity) of symptoms. Depression scale assesses dysphoria, hopelessness, devaluation of life, self-deprecation, lack of interest, anhedonia, and inertia. The anxiety scale assesses autonomic arousal, skeletal muscle effects, situational anxiety, and subjective experience of anxious affect. Finally, the stress scale assesses difficulty relaxing, nervous arousal, easily upset, agitated, irritable, over-reactive, and impatient.

Overall quality of life was assessed using the five-item World Health Organization Well-being Index (WHO-5, Bech et al., 1996). Participants rate each item on a six-point Likert scale based on their feelings over the past two weeks. Higher scores indicate a higher quality of life or level of well-being.

#### 2.3.3. Interoceptive awareness

The Multidimensional Assessment of Interoceptive Awareness-2 (MAIA-2; Mehling et al., 2018) was used to assess interoceptive bodily awareness. It has 37 items, belonging to one of the eight dimensions: noticing, not-distracting, not-worrying, attention regulation, emotional awareness, self-regulation, body listening, and trust. Each item is rated on a six-point scale [“never” (0) to “always” (5)], with higher scores indicating greater bodily awareness. The main reason for including this scale was to explore whether those scoring higher on this scale may benefit more from the CVFLA intervention; or whether the CVFLA intervention increases interoceptive awareness.

### 2.4. CVFLA intervention

The intervention was delivered by a researcher (SC) following a predetermined protocol in up to 10 sessions over 5–10 weeks, depending on the participant’s progress and preference, with each session lasting up to 60 min. Typically, sessions 1–3 focused on introducing and implementing the CVFLA techniques, sessions 4–6 aimed to consolidate previous learning, and sessions 7–9 focused on confirming the re-learning of smell and taste.

**Figure 1:**
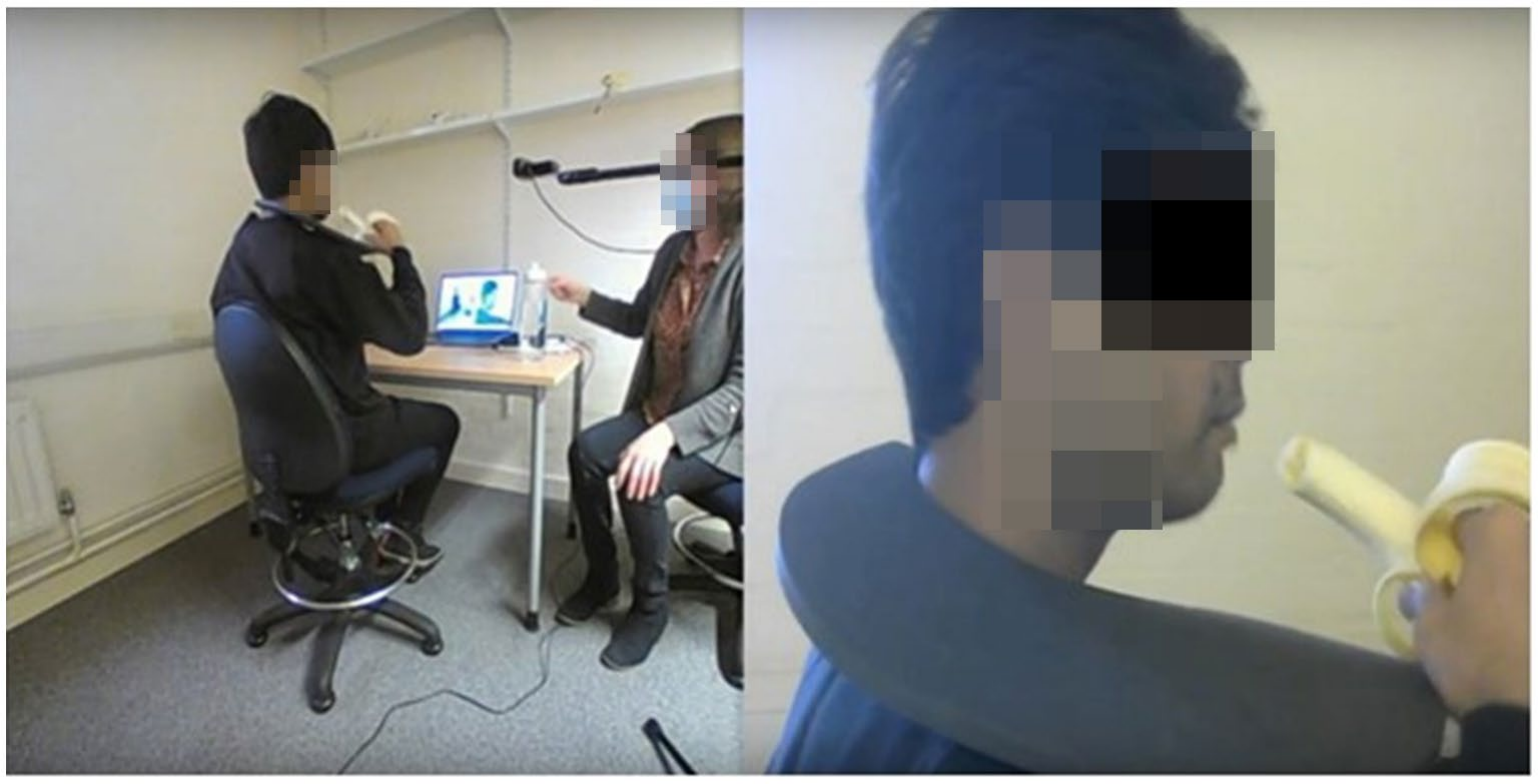
An illustration of the CVLFA set-up and intervention. This image is a screen shot from the computer screen that the participant is watching. The participant observes a real-time video of their actions, via the two webcams being streamed to the computer. The black collar, worn around the neck, blocks the individual’s direct view of their self, meaning the visual information about their actions is now restricted to being ***only*** what they can see on the computer screen. In this illustration, as the banana is eaten, the taste of the banana may be re-learnt since the “taste” of the banana has been learnt previously, and most likely being predicted. The real-time video stream on the computer provides new/additional visual feedback for the participant to learn from.

All CVFLA sessions were conducted with participants sitting on a comfortable chair. During the first CLFLA session, the participant was briefed about the CVFLA set up (Figure 1) with a practical demonstration. A collar was then placed around their neck, and they were asked to observe themselves in real time (with ˜100-ms delay) on the computer screen from two different views: (i) a close-up view to help them focus on the task that they were performing (e.g., smelling or tasting a food item) and (ii) a wide-angle view which showed a broader view of themselves sitting on the chair (Figure 1). If they reported feeling aroused or stressed (or appeared stressed), the demonstration was immediately paused, the collar was removed, the cameras were moved away, and a relaxation exercise (breathing and/or muscle relaxation) was introduced to give them time to recover. Once the participants were comfortable with the practical demonstration and the collar, the session began.

Before each session, the participants were asked to indicate their ability to smell and taste on a scale of 0 to 10, with zero indicating “no smell or taste” and 10 indicating “maximum smell or taste”. This was followed by a breathing and muscle relaxation exercise. Specific smell and taste experiences for any particular food item were then generated in three successive attempts, with each attempt lasting for about 15–25 s. If the participant showed a clear improvement in smell or taste, further attempts were made with another food item in the same category; if no improvement occurred, a different item from a different taste category was presented. Within five taste categories (sweet, sour, salty, umami, and bitter), different food items (e.g., salt, jam, dates, cream crackers, malted biscuits, watercress) were presented; and within each category, the items were clustered by intensity, going from the least to the most intense within the session (e.g., umami - seaweed, soy sauce, bovril, marmite; sour- goji berries, cherries, cranberries; bitter- broccoli, rocket, kale, coffee beans). For the sour category, flavored and plain yoghurt, citrus fruits (grapes, raspberry, satsumas, oranges), apple cider vinegar, candies, lemon, and lime were given depending upon individual’s progress. The food items were presented in a different order for individual sessions and participants depending upon the progress and choice of the participant. However, we consistently started all sessions with a tiny amount of sugar for all participants, regardless of the stage of intervention and progress of the individual, to maintain some consistency. The participants were not blinded to any food item and had been asked in advance for any known allergies and food/smell preferences. Whenever participants reported an unpleasant response (e.g., disgust or stress) to any food items, relaxation exercises were re-introduced to reduce their emotional stress response and/or physical tension. All sessions ended with a breathing or muscle relaxation exercise, as per the participant’s preference.

In addition, during the second and subsequent CVFLA sessions, the participant was also asked to describe any observable changes (from the previous session) in their smell and taste (in addition to indicating their ability to smell and taste on a scale of 0 to 10 as mentioned above for all sessions). These sessions proceeded with taste/flavors based on the participant’s experience from the previous session/week and their comments from the current week, focusing on food and smell items that still needed to be accurately tasted. The number of actual sessions depended upon the individual’s progress. Throughout the sessions, participants’ responses were recorded for identification accuracy and pleasantness/unpleasantness of the item.

### 2.5. Data analysis

As this was a proof-of-concept study with only 15 participants, the data for each participant on all key measures are first presented and summarized descriptively and then analyzed across the entire sample using repeated-measures analysis of variance (ANOVA) to explore the impact of the CVFLA on the primary outcome variable/s (i.e., improvement in the sense of smell and/or taste); the effect sizes where reported are partial eta squared (*η_p_*^2^; the proportion of variance associated with a factor). Next, Pearson’s correlations were used to examine whether the pre- to post-CVFLA changes seen in the primary outcome variables were correlated with any baseline sample characteristics, including age, the duration of smell and taste impairment, various measures of mental health and well-being, and interoceptive awareness. Following the observation of significant associations of post-intervention reduction (improvement) in smell and taste impairment with age, the duration of smell or taste impairment, and the “Noticing” subscale of the MAIA-2 (Interoceptive Awareness), a stepwise regression analysis was run to explore the most robust correlate of the CVFLA-led improvement. Various measures of mental health and well-being, and interoceptive awareness, were also explored for any pre- to post-intervention changes using repeated-measures ANOVAs. Prior to running these analyses, the data properties (skewness, kurtosis) of all variables, including the subjective ratings of smell and taste, were examined and found suitable for parametric statistical procedures. Alpha level for testing the significance of effects was maintained at *p* ≤ 0.05.

All analyses were conducted using the Statistical Package for Social Sciences (for Windows, version 28; IBM, New York, United States).

## 3. Results

### 3.1. Sample characteristics

On average, the sample had moderate-to-severe smell and taste impairment, lasting for about 8 months prior to taking part in this study (see Tables 2, 3). None of the included participants had a neurodegenerative disorder, but one participant had rhinitis (no. 5), and one participant (no. 6) had asthma.

**Table 2:** Sample characteristics.

Sample characteristics	Mean (*SD*)	Range
Age (in years)	43.53 (12.25)	20–62
Duration of smell/taste impairment prior to receiving CVFLA (in days)	236.66 (234.91)	28–817
*Mental health and well-being*		
Depression (DASS-21)	9.13 (7.97)	0–30
Anxiety (DASS-21)	10.80 (10.25)	0–32
Stress (DASS-21)	13.33 (9.96)	0–30
Well-being Index 9 (WHO-5)	13.00 (4.31)	7–21
*Introspective awareness*		
Noticing (MAIA-2)	3.75 (0.86)	1.75–5
Not-distracting ((MAIA-2)	2.14 (1.39)	1.60–4.85
Not-worrying (MAIA-2)	2.87 (0.68)	0.60–3.80
Attention-regulation (MAIA-2)	3.15 (1.24)	1.29–5
Emotional- awareness (MAIA-2)	4.03 (0.81)	2.20–5
Self-regulation (MAIA-2)	3.38 (1.08)	1.50–5
Body-listening (MAIA-2)	2.75 (1.32)	1–5
Trusting (MAIA-2)	3.53 (1.23)	1–5

DASS-21, Depression Anxiety Stress Scale (DASS-21; Lovibond and Lovibond, 1995); WHO-5, World Health Organization Well-being Index (WHO-5; Bech et al., 1996). MAIA-2: Multidimensional Awareness of Introspective Awareness-2 (MAIA-2; Mehling et al., 2018)

**Table 3:** Duration of smell and taste impairment, and subjective ratings of impairment before and after CVFLA for individual participants.

Participant No.	Age (years)	Sex	Duration of smell/taste impairment prior to receiving CVFLA (in days)	Baseline (in days)	Subjective ratings of impairment [scale 0 (none)-to-6 (very severe)]								Pre- to post-CVFLA decrease in impairment across smell and taste (total pre- *minus* total post ratings)
					Smell				Taste				
					Loss		Distortion		Loss		Distortion		
					Pre	Post	Pre	Post	Pre	Post	Pre	Post	
1	31	Female	172*	7	5	2	5	2	5	2	5	2	12
2	36	Female	28	14	5	0	0	0	5	0	0	0	10
3	49	Female	177*	21	4	2	4	3	4	3	4	3	5
4	50	Male	208	7	5	3	5	5	5	1	5	5	6
5	38	Female	207*	14	2	1	5	1	0	0	4	1	8
6	37	Female	207*	21	3	0	3	1	4	0	4	1	12
7	39	Female	50	7	6	0	6	3	5	0	5	2	17
8	59	Female	220*	14	6	2	6	1	1	1	1	1	9
9	60	Male	817*	21	4	2	2	1	1	2	0	1	1
10	41	Male	32	7	4	0	0	0	0	0	6	0	10
11	62	Female	533*	14	5	3	5	3	5	3	2	2	6
12	37	Female	619*	21	2	0	2	0	2	0	2	0	8
13	20	Male	54	7	5	3	5	3	2	1	3	1	7
14	36	Female	172*	21	5	1	5	1	5	1	5	1	16
15	58	Male	54	21	4	1	1	1	4	1	1	1	6

*Participants with impairment for more than 24 weeks.

### 3.2. CVFLA intervention delivery and acceptability

On average, study participants attended about seven intervention sessions (mean = 7.47; SD = 2.56), taking on average about 7 h per person (mean = 389.96 min, SD = 150.93). The intervention was extremely well received, as evident from responses to the feedback interview questions presented in Table 4. All participants found the intervention training to be “generally” or “definitely” useful and enjoyable and believed that it helped them to recover their sense of taste and/or smell. Around one-third (36.37%) of the sample reported that they had practiced the methods and techniques learnt during the sessions outside the sessions (e.g., at home), and they all stated that they would recommend this intervention to other individuals with taste and smell impairment. One person reported concerns regarding body image issues once they began to enjoy food after the fourth CVFLA session. Several participants reported during the last interview that they were skeptical about the intervention and found watching them on camera somewhat uncomfortable initially but were pleasantly surprised with how it helped them to recover their smell and taste. There were no drop-outs due to the CVFLA intervention not being acceptable.

**Table 4:** Post-CVFLA feedback from individual study participants.

Post-CVFLA Feedback (n-11)	Questions				
	Overall, how did you find the CVFLA?	Do you believe the CVFLA has aided in improving your loss and/or distorted sense of taste and/or smell?	Did you find the intervention enjoyable and helpful?	Did you practice the methods and techniques used during the sessions at home or any other place than the lab?	Will you recommend this to other individuals with Taste and Smell impairments?
	Response Options				
	1 = not at all useful	1 = not at all	1 = not at all	1 = not at all	1 = not at all
	2 = not really useful	2 = not really	2 = not really	2 = not really	2 = not really
	3 = yes generally useful	3 = yes generally	3 = yes generally	3 = yes generally	3 = yes generally
	4 = yes definitely useful	4 = yes definitely	4 = yes definitely	4 = yes definitely	4 = yes definitely
Participant no.	Participant responses				
01	4	4	4	1	4
03	3	4	4	1	4
04	4	4	4	4	4
05	3	3	4	1	4
09	4	4	4	2	4
10	3	4	3	4	4
11	4	4	4	4	4
12	4	4	4	4	4
13	3	4	4	2	4
14	4	4	4	2	4
15	4	4	4	2	4

### 3.3. Pre- to post-intervention changes in smell and taste impairment

**Figure 2:**
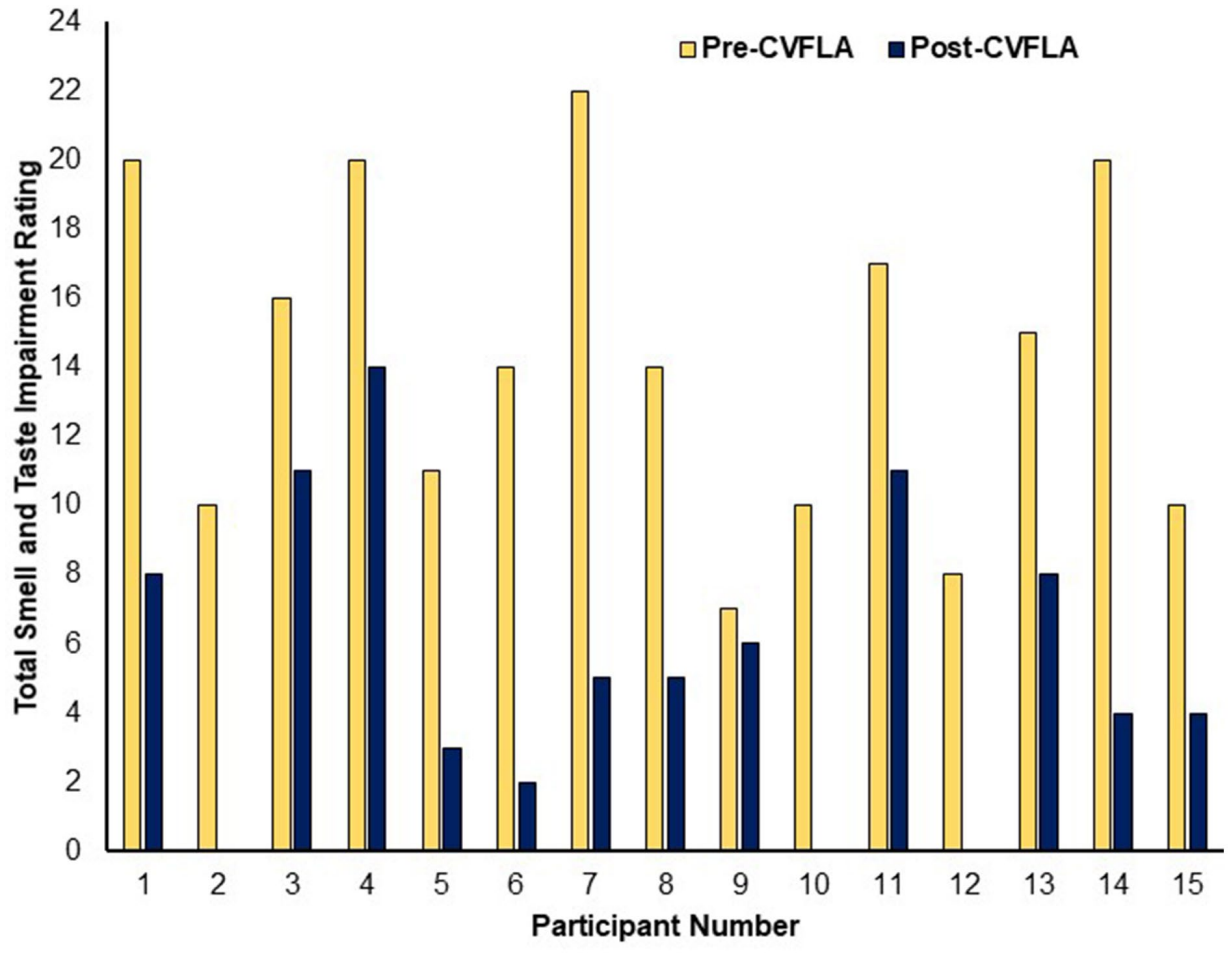
Subjective ratings of total smell and taste impairment before and after the CVFLA intervention.

In post-intervention subjective ratings, relative to the pre-intervention ratings, all participants reported less severe impairment (or no loss) in their sense of smell, 11 participants (of 13 participants with a distorted sense of smell at pre-intervention) reported less severe or no distortion of smell, 11 participants (of 13 participants who had taste impairment) showed a reduction in the severity of taste impairment, and 11 (of 13 participants) showed a less distorted sense of taste (Table 3). All participants, except one, started to report experiencing a positive change in smell and/or taste from the second or third session (session-wise data not presented as the number of sessions varied for individual participants depending on their progress); and each of the 15 participants showed some reduction in total (smell and taste) impairment as assessed by subjective ratings (see Figure 2).

When explored across the entire sample using repeated-measures ANOVAs, there was a significant reduction in subjective ratings of both smell and taste impairment after, compared to before, the CVFLA intervention (all *p* ≤ 0.004), with somewhat larger effect sizes for smell than taste, and for recovery (based on “loss of smell” or “loss of taste ratings”) relative to correction of distorted smell or taste (Table 5). This improvement (total across smell and taste loss and distortion ratings) was correlated negatively with age [*r* = −0.514 (95% CI −0.812, −0.023), *p* = 0.05] and the duration of smell or taste impairment [*r* = −0.529 (95% CI −0.819, −0.002), *p* = 0.04] and positively with pre-intervention scores on the “Noticing” dimension of the MAIA-2 (Interoceptive Awareness) scale [*r* = 0.544 (95% CI 0.043, 0.826), *p* = 0.036]; there was also a trend-level positive association with the Emotional-Awareness dimension of MAIA-2 [*r* = 0.47 (95% CI −0.055, 0.792), *p* = 0.07]. The regression model with these variables as predictors and improvement in smell and taste as the dependent variable was significant (*F* = 5.45, *df* = 1, 14, *p* = 0.036), with a significant effect of the ‘Noticing’ dimension (standardized coefficient β = 0.544, *t* = 2.335, *p* = 0.036); age, the duration of smell or taste impairment, and Emotional-Awareness (MAIA-2) were not significant (all *p* > 0.10). No measure of mental health, well-being, or interoceptive awareness showed a significant difference between pre- and post-CVFLA assessments (all *p* values >0.10).

**Table 5:** Descriptive statistics for subjective ratings of smell and taste impairment and objective (ODOFIN test) assessment of smell and taste identification accuracy before and after the CVFLA intervention and the results of the ANOVAs analyses.

Assessment	Pre-CVFLA (baseline)	Post-CVFLA	ANOVA: Pre- vs. Post-CVFLA comparison		
Subjective ratings of impairment	Mean (*SD*)	Mean (*SD*)	*F (df = 1.14)*	*p*	Effect size (*η_p_^2^*)
Loss of smell	4.33 (1.23)	1.33 (1.17)	72.69	**<0.001**	0.839
Distorted smell	3.60 (2.10)	1.67 (1.45)	21.25	**<0.001**	0.603
Loss of taste	3.20 (1.97)	1.00 (1.07)	18.68	**<0.001**	0.572
Distorted taste	3.13 (1.99)	1.40 (1.30)	11.92	**0.004**	0.460
Total (Smell and Taste) Impairment	14.27 (4.83)	5.40 (4.30)	67.19	**<0.001**	0.828
ODOFIN test for smell and taste identification accuracy^a^	Mean (*SD*)	Mean (*SD*)	*F* (*df* = 1.7)	*p*	Effect size (*η_p_^2^*)
Left nostrils	7.12 (2.36)	10.37 (1.40)	23.20	**0.002**	0.768
Right nostrils	7.37 (2.26)	10.37 (1.19)	12.60	**0.009**	0.643
Both nostrils	7.37 (2.39)	10.75 (1.03)	20.01	**0.003**	0.741
Taste test total^a^	3.12 (0.99)	3.87 (0.35)	4.20	0.08	0.375

a Sample size reduced to 8 due to late arrival of the test kit or missed final in-person follow-up assessment.

**Figure 3:**
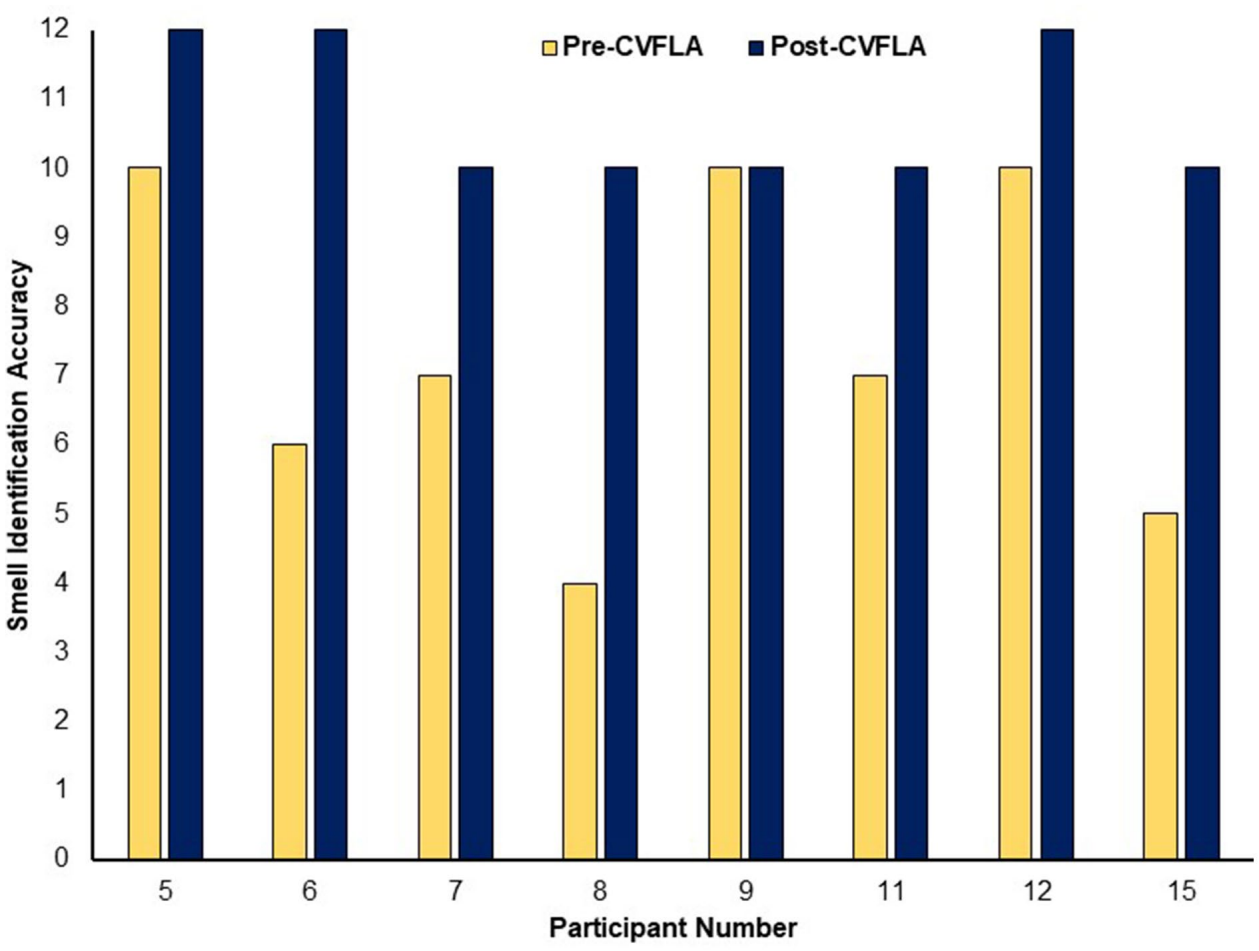
Objective (ODOFIN test) assessment of smell identification accuracy before and after the CVFLA intervention. With the 12 Sniffin’ Sticks test, scores 0–6 indicate anosmia, scores 7–10 indicate hyposmia, and scores 11–12 indicate normosmia.

**Figure 4:**
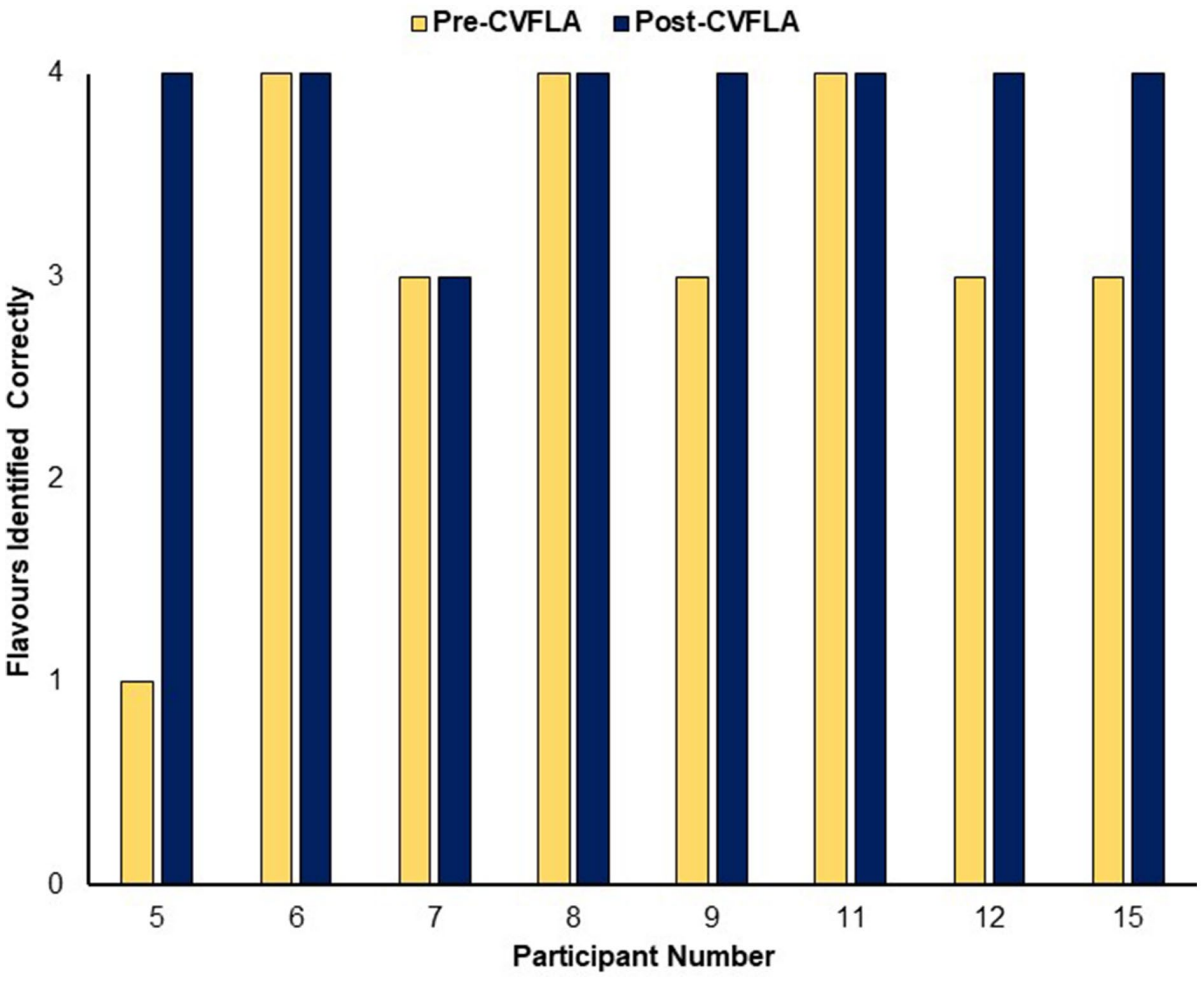
Objective (ODOFIN test) assessment of taste identification accuracy before and after the CVFLA intervention.

An improvement in taste and smell following the intervention was also visible in the smell and taste identification accuracy (ODOFIN test) scores (Figures 3, 4) of 8 participants for whom pre- and post-intervention data were available (unavailable for 7 participants due to late arrival of the test kit or no final in-person follow-up assessment). Exploratory analyses of these data across the entire sample using repeated-measures ANOVAs (Table 5) indicated significantly higher identification accuracy for smells at the post-intervention assessment compared to the pre-intervention assessment (*p* ≤ 0.004) (see Table 5). There was a positive change also for taste identification accuracy, but only at the trend level. Improvements in smell identification correlated in the same direction as noted earlier for subjective ratings but non-significantly (*n = 8*) with symptom duration [*r* = −0.688 (95% CI -0.938, 0.032), *p* = 0.059] and Noticing dimension of the MAIA-2 (Interoceptive Awareness) scale [*r* = 0.504 (95% CI 0.311, 0.892), *p* = 0.203] (no correlation with age, *r* = 0.016).

## 4. Discussion

This was the first study to assess the feasibility and acceptability of a new Camera-Based Visual Feedback Learning Aid (CVFLA) and explore its potential to restore or improve persistent COVID-19-related smell and/or taste impairment. The findings demonstrated that this non-invasive intervention is highly acceptable and can be easily administered even in non-clinical settings, contributing to the accessibility and feasibility of the intervention. The findings also suggested that the intervention could be helpful to people who have COVID-19-related loss or distortion of smell and taste, with relatively stronger benefits in people who scored relatively higher on the “noticing” aspect of interoceptive awareness (assessed with items, such as “I notice changes in my breathing, such as whether it slows down or speeds up.”). The effects of CVFLA seemed somewhat stronger for smell than taste; and for recovery of the lost smell or taste, relative to correction of distorted smell or taste though this might, at least partly, be explained by the sample characteristics (i.e., relatively more severe impairment of smell than taste; and relatively more participants with loss of the sense of smell/taste rather than the distorted sense of smell or taste).

The findings of this proof-of-concept study support the CVFLA as a novel and innovative approach to improving smell and taste that is scalable and may also be preferable to other treatments for taste and smell recovery, such as corticosteroids (Harless and Liang, 2016), which may cause dependency and side-effects in at least a proportion of the users. Furthermore, this approach to improving or correcting smell and taste may also be applied in many different clinical and non-clinical settings, for example, in the context of aging (Delgado-Lima et al., 2023) and neurodegenerative disorders (Hawkes, 2006) where smell and taste alterations are typical problems. However, this was the first study to have tested this intervention in a relatively small number of participants who appeared highly motivated to regain their sense of smell and taste (some people cried with happiness when first reporting improvement during the session). Further studies involving larger samples and appropriate control groups are needed to confirm its potential for recovering or correcting smell and taste in relevant clinical and non-clinical populations.

Concerning the possible mechanisms that might be involved in smell or taste improvement following the CVFLA intervention, one possibility is that it facilitated re-learning of the smell or taste via their correct prediction by the brain (from previous episodic memories of the smell and food items) in response to the visual signals received during the intervention sessions (Clark, 2013; Hutchinson and Barrett, 2019). For example, as shown in Figure 1, the taste of a banana may be re-learnt with additional visual feedback provided to the participant to learn from, since the “taste” of the banana has been learnt previously and is most likely being predicted. Our finding showing a positive relationship between the ‘noticing’ aspect of interoceptive awareness and the degree of improvement suggests that attention and interoceptive awareness may facilitate this effect. There is recent evidence for COVID-19 related anosmia to be associated with higher functional connectivity between the left orbitofrontal cortex and visual association areas, along with greater cerebral blood flow in the hippocampus, insula, and posterior cingulate (Wingrove et al., 2023). Some of these areas may be involved in CVFLA-led benefits given their known roles in episodic memory (hippocampus; Danieli et al., 2023), interoceptive awareness (insula; Craig, 2009; Evrard, 2019), and recall of self-related information (posterior cingulate; Morel et al., 2014). Another factor deserving some comment in the context of our study is the use of breathing exercises during the intervention sessions that may have contributed, at least partly, to the observed smell and taste improvement, given recent evidence for respiration-driven normalization of the olfactory cortex (Gonzalez et al., 2023).

The present study had a number of limitations. First, the study involved only 15 participants and four of these participants did not complete their final in-person post-intervention assessments. Second, it cannot be ruled out that some improvement in taste and smell, especially in participants who had less than eight weeks of impairment, occurred simply with time (independent of 5–10 weeks of receiving CVFLA), although a noticeable improvement was also present in participants who had smell and taste impairment for more than six months, and all participants subjectively reported that the CVFLA intervention was helpful to them. Third, some participants reported practicing smelling and tasting in front of a mirror in between intervention sessions which may have potentially introduced a confound. Fourth, we did not use the complete Sniffin’ Sticks Extended test which may have provided a more detailed assessment of the olfactory function and, in addition, complete pre- and post-CVFLA data on ODOFIN test assessment of smell and taste identification accuracy were available for only 8 of the 15 participants due to late arrival of the test kit or missed final in-person follow-up assessment for various reasons. Lastly, the intervention may be more beneficial for the recovery of smell than taste or, alternatively, the recovery of taste may follow smell recovery. A longer follow-up of the participants in further studies may help to clarify this as well as any secondary effects on mental health that may follow a different time course.

In conclusion, the new CVFLA intervention tested in this proof-of-concept study showed a very high level of acceptability and appeared to be a promising powerful tool to improve smell and taste. Further studies involving larger samples and appropriate control groups are required to confirm the effectiveness of this new intervention in improving smell and/or taste impairment in relevant non-clinical and clinical groups and to examine potential mediators and moderators of its effectiveness.

## Data availability statement

The raw data supporting the conclusions of this article will be made available by the authors, without undue reservation.

## Ethics statement

The studies involving human participants were reviewed and approved by the College of Health, Medicine and Life Sciences Research Ethics Committee (DLS) Brunel University London. The patients/participants provided their written informed consent to participate in this study.

## Author contributions

VK and JB contributed to conceptualization of the study and funding acquisition. SC contributed to research design, participant recruitment, intervention delivery, data acquisition, manuscript creation, and review and editing. KV contributed to data acquisition and scoring, and manuscript review and editing. EA contributed to project administration, and manuscript review and editing. VK contributed to project administration, research design, data analysis, manuscript creation, and review and editing. JB contributed to staff training for intervention delivery. All authors have reviewed the manuscript prior to submission.

## Funding

The study received funding from Brunel University London, and the European Research Development Fund (EDRF) and Learning JBE Ltd. via Anglia Ruskin University. Learning JBE Ltd. owns the patent on the camera-based feedback learning technique used in the study. Learning JBE Ltd. was not involved in the study design, collection, analysis, interpretation of data, the writing of this article or the decision to submit it for publication.

## Conflict of interest

JB was employed by Learning JBE Ltd.

The remaining authors declare that the research was conducted in the absence of any commercial or financial relationships that could be construed as a potential conflict of interest.

## Publisher’s note

All claims expressed in this article are solely those of the authors and do not necessarily represent those of their affiliated organizations, or those of the publisher, the editors and the reviewers. Any product that may be evaluated in this article, or claim that may be made by its manufacturer, is not guaranteed or endorsed by the publisher.
